# Simplified ontologies allowing comparison of developmental mammalian gene expression

**DOI:** 10.1186/gb-2007-8-10-r229

**Published:** 2007-10-25

**Authors:** Adele Kruger, Oliver Hofmann, Piero Carninci, Yoshihide Hayashizaki, Winston Hide

**Affiliations:** 1 South African National Bioinformatics Institute, University of the Western Cape, Bellville 7535, South Africa; 2 Genome Exploration Research Group (Genome Network Project Core Group), RIKEN Genomic Sciences Center (GSC), RIKEN Yokohama Institute, 1-7-22 Suehiro-cho, Tsurumi-ku, Yokohama, Kanagawa, 230-0045, Japan; 3 Genome Science Laboratory, Discovery Research Institute, RIKEN Wako Institute, 2-1 Hirosawa, Wako, Saitama, 351-0198, Japan

## Abstract

Model organisms represent an important resource for understanding the fundamental aspects of mammalian biology. Mapping of biological phenomena between model organisms is complex and if it is to be meaningful, a simplified representation can be a powerful means for comparison. The Developmental eVOC ontologies presented here are simplified orthogonal ontologies describing the temporal and spatial distribution of developmental human and mouse anatomy. We demonstrate the ontologies by identifying genes showing a bias for developmental brain expression in human and mouse.

## Abstract 2

The Developmental eVOC ontologies presented are simplified orthogonal ontologies describing the temporal and spatial distribution of developmental human and mouse anatomy.

## Background

### Ontologies and gene expression

Biological investigation into mammalian biology employs standardized methods of data annotation by consortia such as MGED (Microarray Gene Expression Data Society) and CGAP (Cancer Genome Anatomy Project) or collaborative groups such as the Genome Network Project group at the genome Sciences Centre at RIKEN, Japan [1]. Data generated by these consortia include microarray, CAGE (capped analysis of gene expression), SAGE (serial analysis of gene expression) and MPSS (massively parallel signature sequencing) as well as cDNA and expressed sequence tag (EST) libraries. The diversity of data types offers opportunity to capture several views on concurrent biological events, but without standardization between these platforms and data types, information is lost, reducing the value of comparison between systems. The terminology used to describe data provides a means for the integration of different data types such as EST or CAGE.

An ontology is a commonly used method of standardization in biology. It is often defined as a formal description of entities and the relationships between them, providing a standard vocabulary for the description and representation of terms in a particular domain [2,3]. Given a need and obvious value in the comparison of gene expression between species, anatomical systems and developmental states, we have set out to discover the potential and applicability of such an approach to compare mouse and human systems.

Many anatomical and developmental ontologies have been created, each focusing on their intended organisms. As many as 62 ontologies describing biological and medical aspects of a range of organisms can be obtained from the Open Biomedical Ontologies (OBO) website [4], a system set up to provide well-structured controlled vocabularies of different domains in a single website. The Edinburgh Mouse Atlas Project (EMAP) [5] and Adult Mouse Anatomy (MA) [6] ontologies are the most commonly used ontologies to describe mouse gene expression, representing mouse development and adult mouse with 13,730 and 7,702 terms, respectively. Mouse Genome Informatics (MGI), the most comprehensive mouse resource available, uses both ontologies. Human gene expression, however, can be represented as developmental and adult ontologies by the Edinburgh Human Developmental Anatomy (HUMAT) ontology [7], consisting of 8,316 terms, and the mammalian Foundational Model of Anatomy (FMA) [8], consisting of more than 110,000 terms. Selected terms from the above ontologies have been used to create a cross-species list of terms known as SOFG Anatomy Entry List (SAEL) [9]. Although these ontologies more than adequately describe the anatomical structures of the developing organism, with the exception of SAEL, they are structured as directed acyclic graphs (DAGs), defined as a hierarchy where each term may have more than one parent term [6]. The DAG structure adds to the inherent complexity of the ontologies, hampering efforts to align them between two species, making the process of a comparative study of gene expression events a challenge.

Efforts are being implemented in order to simplify ontologies for gene expression annotation. The Gene Ontology (GO) Consortium's GO slim [10] contains less than 1% of terms in the GO ontologies. GO slim is intended to provide a broad categorization of cDNA libraries or microarray data when the fine-grained resolution of the original GO ontologies are not required. Another set of simplified ontologies are those from eVOC [11]. The core eVOC ontologies consist of four orthogonal ontologies with a strict hierarchical structure to describe human anatomy, histology, development and pathology, currently consisting of 512, 180, 156 and 191 terms, respectively. The aim of the eVOC project is to provide a standardized, simplified representation of gene expression, unifying different types of gene expression data and increasing the power of gene expression queries. The simplified representation achieved by the eVOC ontologies is due to the implementation of multiple orthogonal ontologies with a lower level of granularity than its counterparts.

### Mammalian development

The laboratory mouse is being used as a model organism to study the biology of mammals [12]. The expectation is that these studies will provide insight into the developmental and disease biology of humans, colored by the finding that 99% of mouse genes may have a human ortholog [13], and cDNA libraries can be prepared from very early mouse developmental stages for gene expression analysis.

The study of developmental biology incorporates the identification of both the temporal and spatial expression patterns of genes expressed in the embryo and fetus [14]. It is important to understand developmental gene expression because many genetic disorders originate during this period [13]. Similarities in behavior and expression profiles between cancer cells and embryonic stem cells [15] also fuel the need to investigate developmental biology.

Using mice as model organisms in research requires the need for comparison of resulting data and provides a means to compare mouse data to human data [13]. The cross-species comparison of human and mouse gene expression data can highlight fundamental differences between the two species, impacting on areas as diverse as the effectiveness of therapeutic strategies to the elucidation of the components that determine species.

### Cross-species gene expression comparison

Function of most human genes has been inferred from model organism studies, based on the transitive assumption that genes sharing sequence similarity also share function when conserved across species [16]. The same principle can be applied to gene regulation. The first step is to find not only the orthologs, but the commonly expressed orthologs. We predict that although two genes are orthologous between human and mouse, their expression patterns differ on the temporal and spatial levels, indicating that their regulation may differ between the two species.

The terminology currently used to annotate human and mouse gene expression can be ambiguous [17] among species, which is a result of different ontologies being used to annotate different species. Although the EMAP, MA, HUMAT and FMA ontologies describe the anatomical structures throughout the development of the mouse and human, their complexities complicate the alignment of the anatomy between the two species. With the alignment of terms between a mouse and human ontology, the data mapped to each term become comparable, allowing efficient and accurate comparison of mammalian gene expression. A SAEL-related project, XSPAN [18], is aimed at providing a web tool to enable users to find equivalent terms between ontologies of different species. Although useful, the ontologies used describe only spatial anatomy and are not temporal.

We have attempted to address the issue by developing simplified ontologies that allow the comparison of gene expression between human and mouse on a temporal and spatial level. The distribution of human and mouse anatomy terms across development match the structure of the human adult ontologies that form the core of the eVOC system.

Due to the ambiguous annotation of current gene expression data between human and mouse, and the lack of data mappings accompanying the available ontologies, the ontologies presented here have been developed in concert with semi-automatic mapping and curation of 8,852 human and 1,210 mouse cDNA libraries. We have therefore created a resource of standardized gene expression enabling cross-species comparison of gene expression between mammalian species that is publicly available.

## Results and discussion

### Ontology development

The ontologies were originally created to accommodate requests by the FANTOM3 consortium [19] for a simple mouse ontology that could be used in alignment to the human eVOC ontologies. The FANTOM3 project was a collaborative effort by many international laboratories to analyze the mouse and human transcriptome. The aim was to generate a transcriptional landscape of the mouse genome that led to the evolutionary and comparative developmental analysis in mammals. The ontologies presented here provided the FANTOM3 consortium with a platform to compare the human and mouse transcriptome in the context of mammalian development.

Shared structure between the ontologies ensures effective interoperability on the developmental and species levels. The importance of shared structure between two ontologies becomes apparent when attempting to align them for comparison. If two terms in an ontology are mapped to each other, ontology rules infer that the children terms in each of the ontologies share the same characteristics. For example, if gene X is mapped to 'heart' in a human ontology and gene Y is mapped to 'cardiovascular system' in mouse, we can infer that because 'cardiovascular system' is the parent of 'heart' in both ontologies, gene X and gene Y have an association with respect to their expression in the cardiovascular system although their annotations are not identical. This is especially important when the granularity of annotation in one species is different to that of another.

Terms from the EMAP, MA and HUMAT ontologies have been used to create 28 mouse and 23 human ontologies, representing the 28 Theiler stages and 23 Carnegie stages of mouse and human development, respectively. The 28 Theiler stages represent mouse embryonic, fetal and adult anatomical development, whereas the 23 Carnegie stages represent only human embryonic development. Human adult is represented by the Anatomical System ontology of the eVOC system, upon which the other ontologies are based. The terms from the source ontologies (EMAP, MA and HUMAT) have been mapped to the equivalent term in the developmental eVOC ontologies to ensure interoperability between external ontologies and eVOC. Terms from the mouse have also been mapped to those from human to enable cross-species comparison of the data mapped.

**Figure 1:**
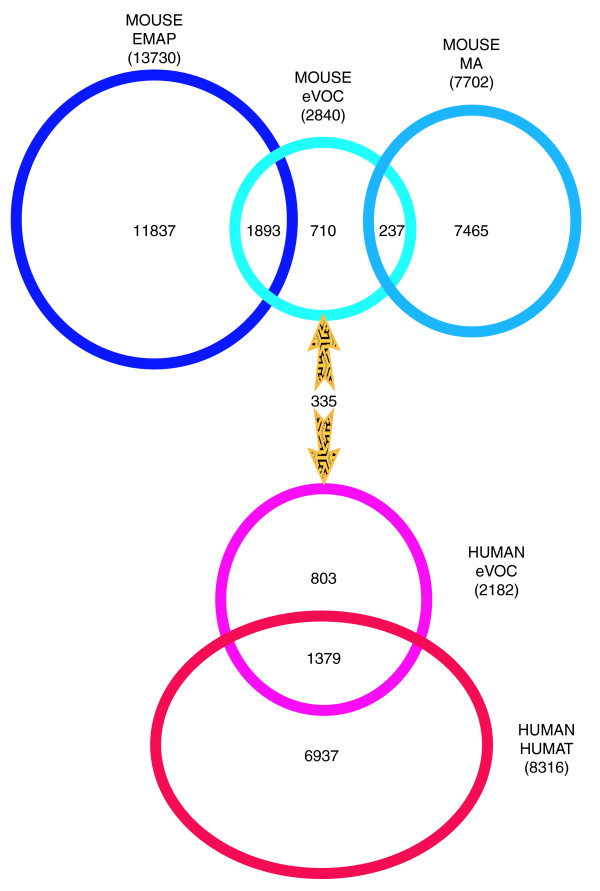
Venn diagram illustrating the integration of mouse and human ontologies represented by the eVOC system. The total number of terms in each ontology is in parentheses. The numbers in each set are the number of terms in the intersection represented by that set. 'Mouse eVOC' represents the 28 individual mouse ontologies and 'Human eVOC' represents the 23 individual human and adult ontologies; therefore, the numbers in parentheses refer to the total number of terms in all the eVOC ontologies for each species. The intersection of the Mouse eVOC with the EMAP and MA ontologies represents the number of terms in Mouse eVOC that have database cross-references to EMAP and MA. Similarly, the intersection of the Human eVOC and HUMAT sets represents the number of Human eVOC terms that map to HUMAT terms. The number within the arrows represents the number of mapped human and mouse eVOC terms.

The integration of the ontologies is described in Figure 1, where 'Mouse eVOC' refers to the individual mouse ontologies and 'Human eVOC' refers to the individual human ontologies (including the adult human ontology). The EMAP and MA ontologies represent mouse pre- and post-natal developmental anatomical structures, respectively, and, therefore, exhibit no commonality. The mouse developmental eVOC ontologies integrate the two ontologies by containing terms from, and mappings to, both the EMAP and MA ontologies. Of the 2,840 terms in the individual mouse ontologies, 1,893 and 237 map to EMAP and MA, respectively. The human developmental eVOC ontology is an untangled version of the HUMAT ontology and has one-to-one mappings to the mouse developmental ontology, providing a link between the terms and data mappings between the mouse and human ontologies.

The presence of species-specific anatomical structures posed a challenge when aligning the mouse and human terms. An obvious example is the presence of a tail in mouse but not in human. We decided that there would simply be no mapping between the two terms. Further challenges involved structures such as paw and hand. The two terms cannot be made identical because it is incorrect to refer to the anterior appendage of a mouse as a hand. However, due to the fact that the mouse paw and human hand share functional similarities, the two terms are not identical, but are mapped to each other based on functional equivalence.

In order to provide simplified ontologies, the 28 mouse and 23 human ontologies were merged to create two ontologies - one for each species. In addition, a Theiler Stage ontology was created that represents the Theiler stages of mouse development. The human stage ontology is represented by the current eVOC Development Stage. A cross-product of two terms (one from the merged and one from the stage ontology) for a species can, therefore, represent any anatomical structure at any stage of development.

**Figure 2:**
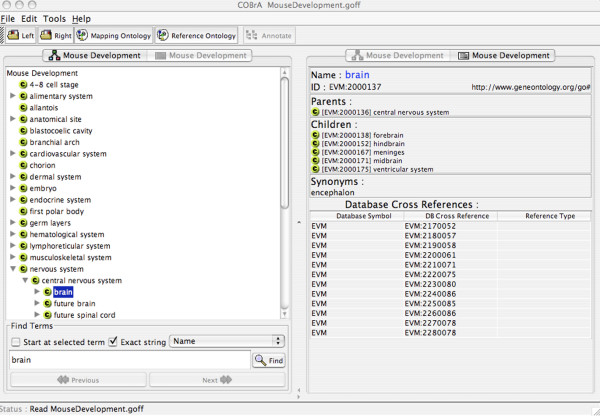
Screenshot of the Mouse Development ontology, visualized in COBrA. The left panel shows the hierarchy of the ontology, with 'brain' as the highlighted term. The right panel lists the 12 database cross-references mapped to 'brain', representing the accession of 'brain' in each of the 12 individual ontologies.

**Figure 3:**
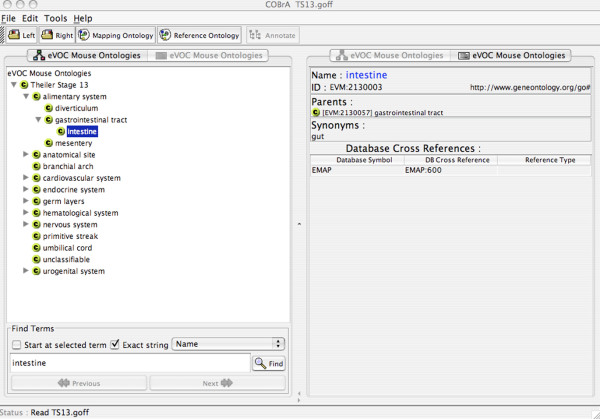
Screenshot of the individual Theiler Stage 13 ontology, visualized in COBrA The left panel displays the ontology with terms of anatomical structures occurring only in Theiler stage 13 of mouse development. The right panel lists the accession of the equivalent term in the external ontology as a database cross-reference.

The relationship between the developmental mouse and individual ontologies is illustrated in Figure 2, where the term 'brain' is mapped to 12 terms in the individual ontologies and, therefore, occurs in 12 of the 28 Theiler stages. All terms in the individual ontologies that are derived from EMAP or MA for mouse, and HUMAT for human are mapped to the corresponding term by adding the term's accession from the external ontology as a database cross-reference in the eVOC ontologies. Figure 3 shows that the database cross-reference is the accession of the EMAP term, indicating that 'intestine' of the 'Theiler Stage 13' ontology is equivalent to the term represented by 'EMAP:600'. This feature allows cross-communication, and thereby integration, of the EMAP, MA, HUMAT and eVOC ontologies.

The ontologies presented here are simplified versions of existing human and mouse developmental and adult ontologies, containing 1,670 and 2,840 terms, respectively. Table 1 shows the number of terms and database cross-references for the individual mouse and human ontologies. The Theiler Stage 4 ontology contains 12 terms and has 9 mappings to the EMAP ontology. The mouse and human stages have been aligned in the table, showing that mouse Theiler stage 4 is equivalent to human Carnegie stage 3, based on morphological similarities during development [20]. The Carnegie Stage 3 ontology contains 13 terms and has 11 mappings to the HUMAT ontology. The difference in the number of ontology terms and external references is attributed to the addition of terms to maintain the standard structure of the eVOC system. In this example, the term 'germ layers' is in the eVOC ontologies, but not in the EMAP or HUMAT ontologies. Many eVOC terms are mapped to more than one term in the external referencing ontology as an artifact of the simplification of the ontologies, resulting in a one-to-many relationship between eVOC and its reference ontology. For example, 'myocardium' at Theiler stage 12 in the eVOC ontologies is mapped to five EMAP identifiers. Each EMAP identifier references a cardiac muscle, but at a different location. eVOC does not distinguish between cardiac muscle of the common atrial chamber (EMAP:337) and cardiac muscle of the rostral half of the bulbus cordis (EMAP:330). Compared to their counterparts, the Developmental eVOC ontologies represent 22% of both the human HUMAT and mouse EMAP ontologies, with the only relationship between the terms being 'IS_A'. Note that relationships within the eVOC ontologies indicate only an association between parent and child term and do not systematically distinguish between is_a or part_of relationships. As eVOC moves to adopt relationship types from the OBO Relation Ontology [21], relations will be reviewed and curated. Using a principle of data-driven development, eVOC terms are added at an annotator's request, resulting in a dynamic vocabulary describing gene expression.

**Table 1:** Statistics of the individual developmental eVOC ontologies, representing the alignment between human and mouse stages

Theiler stage	Mouse terms	External reference	Carnegie stage	Human terms	External reference
1	6	4	1	5	4
2	5	3	2	5	4
3	6	4			
4	12	9	3	13	11
5	9	6			
6	10	7	4	10	8
7	11	9			
8	12	10	5a	10	8
			5b	11	10
			5c	9	8
9	14	14	6a	14	16
			6b	19	18
10	14	18	7	20	17
11	32	29	8	22	19
12	56	63	9	52	54
13	55	64	10	60	80
14	67	85	11	72	92
15	80	109	12	80	98
16	93	128	13	103	131
17	103	137	14	122	149
18	116	155	15	131	165
19	134	173	16	155	178
20	157	171	17	170	184
21	193	239	18	188	223
			19	199	237
22	209	299	20	200	237
23	216	303			
24	226	316			
25	234	339			
26	238	348			
27	266	0			
28	266	246	Adult	512	

Total	2,840	3,288		2,049	1,951

The first three columns display the individual mouse ontologies, the number of terms in each ontology, and the number of external references of each. The last three columns display the individual human ontologies, the number of terms, and the number of external references of each. The external references refer to the EMAP and MA ontologies for mouse, and to HUMAT for human. The alignment of the rows between the mouse and human ontologies represents the alignment of the Theiler and Carnegie stages of development based on morphological similarities. For example, the Theiler Stage 4 ontology contains 12 terms and has 9 mappings to the EMAP ontology. Mouse Theiler Stage 4 is equivalent to human Carnegie Stage 3. The Carnegie Stage 3 ontology contains 13 terms and has 11 mappings to terms from the HUMAT ontology.

### Data mapping

The resources providing ontologies to annotate gene expression do not always provide the data themselves. In order to obtain mouse and human data, one would have to search separate databases for each species. An example of this would be searching MGI for mouse gene expression data, and ArrayExpress for human. Apart form having to access different databases to obtain data, the terminology used to describe the data is ambiguous and differs in the level of granularity, impacting on the accuracy of inter-species data comparison. The ontology terms have, therefore, been used to annotate 8,852 human and 1,210 mouse cDNA libraries from CGAP [22].

The mapping process revealed inconsistencies in the annotation of the human and mouse CGAP cDNA libraries, requiring manual intervention and emphasizing the need for a standardized annotation. All genes associated with the libraries have been extracted by association through UniGene. A gene was considered to be associated with a cDNA library if at least one EST was evident for the gene in a particular library. The result is a set of 21,152 human and 24,047 mouse genes from UniGene that are represented by CGAP cDNA libraries and annotated with eVOC terms, and represent the set of human and mouse genes for which there is expression evidence. CGAP represents an ascertainment bias where there is a strong over-representation for cancer genes, and, therefore, future efforts for this research will include obtaining a well-represented, evenly distributed dataset of human and mouse gene expression. The list of human and mouse orthologs were extracted from HomoloGene to represent the 16,324 human-mouse orthologs. Two genes were considered to be orthologs if they shared the same HomoloGene group identifier.

### Data mining

Genes may be categorized according to their eVOC annotation on a spatial or temporal level, or a combination of both. An example of this would be genes expressed in the heart at Theiler stage 26 for mouse. For the purposes of this study, we searched for human-mouse orthologs that are expressed in the normal postnatal and developmental brain of both species, where a gene is classified as normal if its originating library was annotated as 'normal'. Research involving gene expression of the brain aims at identifying causes of psychological and neurological diseases, many of which originate during development. With the use of mice as model organisms in this kind of research, it is important to identify genes that are co-expressed in human and mouse on the temporal and spatial levels. The results of our analysis show that of the available 16,324 human-mouse orthologs, 14,434 can be found in CGAP libraries for both human and mouse. When looking at brain gene expression, we could segregate genes according to their spatial and temporal expression patterns. We found that of all the orthologs expressed in the brain, 10,980 genes were expressed in the post-natal brain of both species whereas 1,692 genes were expressed in the developing brain of both species. Of these two sets of genes, 90 genes were found to have biased expression for developmental brain (Table 2) where developmentally biased genes are those that are expressed during development and not the post-natal organism in either human, mouse or both species (see Additional data file 1 for illustration). The 9,378 genes found to have a bias for post-natal brain gene expression can be found in Additional data file 2. It is important to note that only genes whose orthologs also have expression evidence were considered for analysis. This small number of genes found to be biased for expression during brain development in both species may be a result of data-bias due to the difficulty involved in accessing developmental libraries. Our future efforts will include expanding the data platforms to provide data that are representative of the biology. This analysis does, however, demonstrate the usefulness of the ontologies in performing cross-species gene expression analyses.

**Table 2:** Genes showing developmental expression bias in human and mouse brain

HomoloGene group identifier	Human Entrez Gene ID	Human Entrez Gene symbol	Mouse Entrez Gene ID	Mouse Entrez Gene Symbol
32	435	ASL	109900	Asl
268	5805	PTS	19286	Pts
413	353	APRT	11821	Aprt
1028	1606	DGKA	13139	Dgka
1290	9275	BCL7B	12054	Bcl7b
1330	857	CAV1	12389	Cav1
1368	1054	CEBPG	12611	Cebpg
1871	4760	NEUROD1	18012	Neurod1
1933	5050	PAFAH1B3	18476	Pafah1b3
2212	6182	MRPL12	56282	Mrpl12
2593	7913	DEK	110052	Dek
2880	8835	SOCS2	216233	Socs2
3476	9197	SLC33A1	11416	Slc33a1
4397	8971	H1FX	243529	H1fx
4983	10991	SLC38A3	76257	Slc38a3
6535	11062	DUS4L	71916	Dus4l
7199	11054	OGFR	72075	Ogfr
7291	10683	DLL3	13389	Dll3
7500	5806	PTX3	19288	Ptx3
7516	389075	RESP18	19711	Resp18
7667	1154	CISH	12700	Cish
7717	24147	FJX1	14221	Fjx1
7922	6150	MRPL23	19935	Mrpl23
9120	25851	DKFZP434B0335	70381	2210010N04Rik
9355	51637	C14orf166	68045	2700060E02Rik
9813	55627	FLJ20297	77626	4122402O22Rik
10026	55172	C14orf104	109065	1110034A24Rik
10494	58516	FAM60A	56306	Tera
10518	84273	C4orf14	56412	2610024G14Rik
10663	57171	DOLPP1	57170	Dolpp1
10695	57120	GOPC	94221	Gopc
10774	57045	TWSG1	65960	Twsg1
11653	79730	FLJ14001	70918	4921525L17Rik
11920	84303	CHCHD6	66098	Chchd6
11980	84262	MGC10911	66506	1810042K04Rik
12021	84557	MAP1LC3A	66734	Map1lc3a
12418	124056	NOXO1	71893	Noxo1
12444	84902	FLJ14640	72140	2610507L03Rik
12993	84217	ZMYND12	332934	Zmynd12
14128	91107	TRIM47	217333	Trim47
14157	90416	CCDC32	269336	Ccdc32
14180	115294	PCMTD1	319263	Pcmtd1
14667	113510	HEL308	191578	Hel308
15843	79591	C10orf76	71617	9130011E15Rik
16890	399664	RKHD1	237400	Rkhd1
17078	387914	TMEM46	219134	Tmem46
17523	115290	FBXO17	50760	Fbxo17
18123	140730	RIMS4	241770	Rims4
18833	143678	LOC143678	75641	1700029I15Rik
18903	440193	KIAA1509	68339	0610010D24Rik
19028	146167	LOC146167	234788	Gm587
20549	4324	MMP15	17388	Mmp15
21334	10912	GADD45G	23882	Gadd45g
22818	29850	TRPM5	56843	Trpm5
24848	266629	SEC14L3	380683	RP23-81P12.8
26702	93109	TMEM44	224090	Tmem44
27813	84865	FLJ14397	243510	A230058J24Rik
31656	27000	ZRF1	22791	Dnajc2
32293	51018	CGI-115	67223	2810430M08Rik
32331	51776	ZAK	65964	B230120H23Rik
32546	64410	KLHL25	207952	Klhl25
32633	136647	C7orf11	66308	2810021B07Rik
35002	93082	LINCR	214854	Lincr
37917	1293	COL6A3	12835	Col6a3
40668	9646	SH2BP1	22083	Sh2bp1
40859	27166	PX19	66494	2610524G07Rik
41703	118881	COMTD1	69156	Comtd1
45198	65117	FLJ11021	208606	1500011J06Rik
45867	139189	DGKK	331374	Dgkk
46116	401399	LOC401399	101359	D330027H18Rik
49899	143282	C10orf13	72514	2610306H15Rik
49970	83879	CDCA7	66953	Cdca7
55434	1289	COL5A1	12831	Col5a1
55599	669	BPGM	12183	Bpgm
55918	6882	TAF11	68776	Taf11
56005	6328	SCN3A	20269	Scn3a
56571	26503	SLC17A5	235504	Slc17a5
56774	54751	FBLIM1	74202	Fblim1
64353	126374	WTIP	101543	Wtip
65280	286128	ZFP41	22701	Zfp41
65318	23361	ZNF629	320683	Zfp629
65328	7559	ZNF12	231866	Zfp12
68420	9559	VPS26A	30930	Vps26
68934	57016	AKR1B10	14187	Akr1b8
68973	1663	DDX11	320209	Ddx11
68998	170302	ARX	11878	Arx
78698	387876	LOC387876	380653	Gm872
81871	56751	BARHL1	54422	Barhl1
82250	150678	MYEOV2	66915	Myeov2
84799	22835	ZFP30	22693	Zfp30

The table lists the HomoloGene group identifier, Entrez Gene identifier and gene symbol of the 90 human-mouse orthologs found to have an expression bias towards the embryonic and fetal stages of brain development, without expression during postnatal development. Genes were considered for analysis only if they have an ortholog, and if the ortholog also has expression evidence based on eVOC annotation.

The GO categories that are highly associated with the 90 genes biased for developmental brain expression were extracted with the use of the DAVID bioinformatics resource [18]. The human representatives of the human-mouse orthologs cluster with GO terms such as 'nervous system development' and 'cell differentiation', suggesting a shared role for development of the mammalian brain, and, therefore, may be potential targets for the analysis in neurological diseases. Given the existence of ascertainment bias on these kinds of data, it was still surprising to see how many genes passed the stringent selection criteria. Searching the Online Mendelian Inheritance of Man (OMIM) database implicated some of the 90 genes, such as *GOPC*, *ARX *and *DEK*, in diseases such as astrocytoma, lissencephaly and leukemia.

To assess the similarity in expression across major human and mouse tissues other than brain, the expression profiles of the 90 genes with bias for developmental expression were determined for developmental and adult expression in the following tissues: female reproductive system, heart, kidney, liver, lung, male reproductive system and stem cell. These tissues were chosen based on the availability of data for each tissue in the developmental and adult categories. For each ortholog-pair, we determined the correlation between their expression profiles (Additional data file 3). We found that, according to the cDNA libraries, one mouse gene was found to be expressed in all the tissues in both post-natal and development (*Twsg1*), and three mouse genes were expressed only in the mouse brain (*Resp18*,*Gm872*,*Barhl1*) as opposed to all other tissues (see Additional data file 4 for expression profile). The highest correlation score between an ortholog-pair is 0.646 (HomoloGene identifier: 27813), having identical expression profiles during development (expressed in liver and stem cell), but differing during post-natal expression (expression in mouse heart, kidney and stem cell but not in their human counterparts). The correlations observed suggest that the expression profiles of orthologs across these major tissues are only partially conserved between human and mouse. This finding strengthens our understanding of orthologous gene expression in that although two genes are orthologs, they do not share temporal and spatial expression patterns and, therefore, probably do not share a majority of their regulatory modules [23].

Developmental gene expression may be subdivided into embryonic and fetal expression, which in turn may be categorized further according to the Theiler and Carnegie stages for mouse and human, allowing a high-resolution investigation of gene expression profiles between the two species. This stage by-stage expression profile for human and mouse will allow investigation into common regulatory elements of co-developmentally expressed genes and give new insight into the characterization of the normal mammalian developmental program.

## Conclusion

The developmental mouse ontologies were developed in collaboration with the FANTOM3 consortium to have the same structure and format as the existing human eVOC ontologies to enable the comparison of developmental expression data between human and mouse. The developmental ontologies have been constructed by integrating EMAP, MA, the developmental Human Anatomy and the human adult eVOC ontologies. The re-organization of existing ontological systems under a uniform format allows the consistent integration and querying of expression data from both human and mouse databases, creating a cross-species query platform with one-to-one mappings between terms within the human and mouse ontologies.

The ontologies have been used to map human and mouse gene expression events, and can be used to identify differential gene expression profiles between the two species. In future, the ontologies presented here will be used to investigate the transcriptional regulation of genes according to their characteristics based on developmental stage, tissue and pathological expression profiles, providing insight into the mechanisms involved in the differential regulation of genes across mammalian development.

## Materials and methods

### Ontology development

The ontologies were constructed using the COBrA [24] and DAG-edit [25] ontology editors. Each term has a unique accession identifier with 'EVM' as the namespace for mouse and 'EV' for human, followed by seven numbers. This is consistent with the rules defined by the GO consortium [26].

Using the human adult eVOC anatomical system ontology as a template, terms from the Theiler stage 26 (mouse developmental stage immediately prior to birth) section of the EMAP ontology were inserted to create the Theiler Stage 26 developmental eVOC mouse ontology. Proceeding from Theiler stage 26 to Theiler stage 1, each stage was used as a template for the next stage and any term not occurring at that specific stage, using EMAP as reference, was removed. Similarly, if a term occurred in EMAP that was not present in the previous stage, it was added to the ontology. The result is a set of 26 ontologies, one for each Theiler stage of mouse development, with many terms appearing and disappearing throughout the ontologies according to changes of anatomy during mouse development.

The Theiler Stage 28 (adult mouse) ontology was constructed in the same way as the developmental ontologies, using the MA ontology as a reference. A previously unavailable Theiler Stage 27 ontology was developed by comparing Theiler stage 26 and Theiler stage 28. Any terms that differed between the two stages were manually curated and included or removed in Theiler stage 27 as needed. The Theiler Stage 27 ontology therefore represents all immature, post-natal anatomical structures. Theiler Stage 28 ontology terms have been mapped to the adult human eVOC terms by using the human eVOC accession identifiers as database cross-references in the mouse ontology. Similarly, the EMAP accession number for each term was mapped to the developmental mouse ontologies. The result is a set of 28 ontologies that are an untangled form of the EMAP and MA ontologies, with mappings between them.

A set of human developmental ontologies were created by using the same method as was used for mouse. The reference ontologies for human development were the HUMAT ontologies, which describe the first 23 Carnegie stages of development, classified according to morphological characteristics.

The 28 mouse and 23 human ontologies were merged into two ontologies - one for mouse and one for human. Each merged ontology (named Mouse Development and Human Development) contains all terms present in the individual ontologies. A Theiler Stage ontology was created for mouse, which contains all 28 Theiler stages categorized into embryo, fetus or adult. The existing eVOC Development Stage ontology serves as the human equivalent of the mouse Theiler Stage ontology. The Mouse Development, Human Development, Theiler Stage and the existing Development Stage ontologies form the core of the Developmental eVOC ontologies.

### Data mapping

Mouse and human cDNA libraries were obtained from the publicly available CGAP resource and mapped (semi-automated) to the entire set of eVOC ontologies. The eVOC ontologies consist of Anatomical system, Cell type, Developmental stage, Pathology, Associated with, Treatment, Tissue preparation, Experimental technique, Pooling and Microarray platform. The 'age' annotation of the mouse CGAP libraries was manually checked against the Gene Expression Database (version 3.41) [27] to determine the Theiler stage of each library. Due to the lack of a resource providing the Carnegie stage annotation for cDNA libraries, the human cDNA libraries were annotated according to the age annotation originally provided by CGAP. Genes associated with each mouse and human cDNA library were obtained from NCBI's UniGene [28]. A list of human-mouse orthologs were obtained from HomoloGene (build 53) [29].

### Data mining

The genes were filtered according to the presence or absence of expression evidence and homology. A gene passed the selection criteria if it has an ortholog and if both genes in the ortholog pair have eVOC-annotated expression. According to eVOC annotation, genes were categorized into those that showed expression in normal adult brain and those expressed in normal developmental brain, many genes appearing in more than one category. Genes expressed in normal adult brain were subtracted from those with expression in normal developmental brain to establish genes whose expression in the brain occurs only during development. The expression profiles of the developmentally biased genes annotated to female reproductive system, heart, kidney, liver, lung, male reproductive system and stem cell for post-natal and developmental expression were determined according to the eVOC annotation of the cDNA libraries, and the correlation coefficient of the ortholog-pairs were calculated.

### Availability

The mouse eVOC ontologies, their mappings and the datasets referred to in this manuscript are available under a FreeBSD-style license at the eVOC website [30].

## Abbreviations

CAGE, capped analysis of gene expression; CGAP, Cancer Genome Anatomy Project; DAG, directed acyclic graph; EMAP, Edinburgh Mouse Atlas Project; EST, expressed sequence tag; FMA, Foundational Model Of Anatomy; GO, Gene Ontology; HUMAT, Edinburgh Human Developmental Anatomy; MA, Adult Mouse Anatomy; MGI, Mouse Genome Informatics; OBO, Open Biomedical Ontologies; SAEL, SOFG Anatomy Entry List.

## Authors' contributions

AK was responsible for ontology development and integration, data mapping, data mining and drafting the manuscript. OH helped with ontology development and integration between the human and mouse ontologies. PC and YH drove development requirements for the study. WH was responsible for study design and revised the manuscript. All authors read and approved the final manuscript.

## Additional data files

The following additional data are available with the online version of this paper. Additional data file 1 is a diagram illustrating the sets of genes analyzed for developmental brain expression bias. Additional data file 2 is a table listing genes not showing developmental expression bias in human and mouse brain. Additional data file 3 is a table listing the correlation coefficients of the 90 genes showing bias for developmental expression in the human and mouse brain. Additional data file 4 shows the expression profiles of the 90 genes showing bias for developmental expression across major human and mouse tissues in the form of a binary pseudoarray.

## Supplementary Material

Additional data file 1 Genes for human and mouse grouped together if they are expressed in post-natal or developmental brain, respectively. The intersection between the human and mouse developmental brain genes represent those genes showing common expression in the two species. Subtracting genes commonly expressed in human and mouse post-natal brain determines those genes that show developmental restriction in either human, mouse or both species. Click here for file

Additional data file 2 The table lists the Entrez Gene identifier and gene symbol of the 9,378 human-mouse orthologs found not to have an expression bias towards the embryonic and fetal stages of brain development. Genes were considered for analysis only if they have an ortholog, and if the ortholog also has expression evidence based on eVOC annotation. Click here for file

Additional data file 3 The table lists the HomoloGene group identifier, Human Entrez Gene identifier, Human Entrez gene symbol, Mouse Entrez Gene identifier, Mouse Entrez gene symbol and the correlation coefficient between the expression profiles of the genes in each species. Click here for file

Additional data file 4 The tissues represented are female reproductive system, heart, kidney, liver, lung, male reproductive system and stem cell for both post-natal and developmental expression. The table lists the HomoloGene group identifier, Entrez Gene identifier and Entrez gene symbol for human and mouse, as well as the species each row represents. Values in the table are 1 if the genes (in rows) are expressed in the given tissues (in columns) and 0 if the genes are not found to be expressed in the tissues. Click here for file
